# Equivalence between solar irradiance and solar simulators in aging tests of sunglasses

**DOI:** 10.1186/s12938-016-0209-7

**Published:** 2016-08-26

**Authors:** Mauro Masili, Liliane Ventura

**Affiliations:** Electrical Engineering Department, Engineering School of São Carlos, University of São Paulo, Av. Trabalhador Sãocarlense 400, São Carlos, SP 13566-590 Brazil

**Keywords:** Solar resistance testing for sunglasses, Sunglasses aging test, Sunglasses standards, Ultraviolet A and B protection for sunglasses, UV ocular protection

## Abstract

**Background:**

This work is part of a broader research that focuses on ocular health. Three outlines are the basis of the pyramid that comprehend the research as a whole: authors’ previous work, which has provided the public to self-check their own sunglasses regarding the ultraviolet protection compatible to their category; Brazilian national survey in order to improve nationalization of sunglasses standards; and studies conducted on revisiting requirements of worldwide sunglasses standards, in which this work is inserted. It is still controversial on the literature the ultraviolet (UV) radiation effects on the ocular media, but the World Health Organization has established safe limits on the exposure of eyes to UV radiation based on the studies reported in literature. Sunglasses play an important role in providing safety, and their lenses should provide adequate UV filters. Regarding UV protection for ocular media, the resistance-to-irradiance test for sunglasses under many national standards requires irradiating lenses for 50 uninterrupted hours with a 450 W solar simulator. This artificial aging test may provide a corresponding evaluation of exposure to the sun.

**Methods:**

Calculating the direct and diffuse solar irradiance at a vertical surface and the corresponding radiant exposure for the entire year, we compare the latter with the 50-h radiant exposure of a 450 W xenon arc lamp from a solar simulator required by national standards.

**Results:**

Our calculations indicate that this stress test is ineffective in its present form.

**Conclusions:**

We provide evidence of the need to re-evaluate the parameters of the tests to establish appropriate safe limits for UV irradiance.

**Significance:**

This work is potentially significant for scientists and legislators in the field of sunglasses standards to improve the requirements of sunglasses quality and safety.

## Background

Ocular health is a serious concern worldwide, but particularly in tropical countries where UV indexes are extremely high in summer and still very high in the winter compared to countries that are farther apart from the tropics. In most countries in the southern hemisphere, and specifically in Brazil, a continental sized tropical country, sunglasses standards are not quite appropriate for the ultraviolet conditions, as well as for the people’s behavior profile about UV protection, and public should be more aware about ultraviolet protection as a whole. The authors of this work have been conducting researches in order to bridge these gaps. Three outlines are the basis of the pyramid that comprehends the research as a whole: (1) authors’ previous work [1], which has provided the public to self-check their own sunglasses regarding the ultraviolet protection compatible to their category. This has allowed population to self-test their own sunglasses for free and in an easy way to find out in 30 s whether their sunglasses are adequate or inappropriate to be worn by the Brazilian standard limits; (2) Brazilian national survey [2] has improved information such as how many daily hours Brazilians wear sunglasses, in which period of the day and season, in which are the environments most popularly worn, what kind of sunglasses are mostly purchased, and so forth. This information provides parameters for nationalization of sunglasses standards, such as how long sunglasses should last in such community; (3) studies conducted on revisiting requirements of worldwide sunglasses standards, such as the UV protection range extended to 400 nm in 2013 in Brazil as part of our researches. This work is a continuation of these researches.

According to the International Commission on Non-Ionizing Radiation Protection (ICNIRP), ultraviolet (UV) radiation constitutes the portion of the electromagnetic spectrum spanning from 100 to 400 nm [3]. The International Commission on Illumination (CIE—Commission Internationale de l’Eclairage) [4, 5] subsequently split the UV spectrum into three important spectral bands with respect to the effects of UV radiation on biological systems. These bands are widely known as UV-C (100–280 nm), UV-B (280–315 nm), and UV-A (315–380 nm or 400 nm, depending on the standard).

Investigations on UV radiation incident upon the eyes have noted pathological modifications to the cornea and to the internal structures of the eye [6, 7]. The possible effects include edema, pterygium, lens opacity (cataract), and retina damage [8, 9].

It is well known that sunglasses should provide filters for protection against UV radiation. National and regional standards [10–14] for the sunglasses industry require that sunglasses provide levels of protection linked to the luminous transmittance, i.e., on the category of lenses. The Australian/New Zealand standard [11], the first one for general use sunglasses, set a UV wavelength range from 280 to 400 nm. The 2013 version of Brazilian standard extended the upper limit of the UV-A range from 380 to 400 nm, becoming more consistent with the Australian/New Zealand standard [11], as Brazil, Australia, and New Zealand share greater risk of a higher UV dose [15]. However, the current Brazilian standard, NBR ISO 12312-1:2015 [10], which replaced the NBR 15111:2013, has returned the UV-A upper limit to 380 nm. In a recent work [2], the authors emphasized the importance of considering the UV-A limit of 400 nm for UV-protecting filters based on the radiant exposure (in J m^−2^) on the eye’s surface.

It is also important to understand the lifetime of the optical properties of sunglasses. The exposure of sunglasses to the sun may deteriorate their UV protection and alter the category under which they are classified (lenses may become lighter when overexposed to the sun) over time. Moreover, Chou, Dain, and, Cheng [16] recently showed that transmittance is not the only factor effected by UV radiation exposure. They showed that exposure of lenses to high levels of UV radiation diminishes the impact resistance of lenses. Thus, it should be a requirement that both the transmittance and the impact tests should be performed subsequently to the aging test of the lenses.

### Aging tests of sunglasses lenses

One of the requirements of the Brazilian standard NBR ISO 12312-1:2015 and other standards is to perform a test in which sunglasses are irradiated by a solar simulator for a specified period. The UV protection provided by the sunglasses prior to exposure to UV radiation is then compared to their UV protection capabilities following exposure in the solar simulator. This test provides a measure of any change in the UV protection as a result of exposure of the sunglasses to the sun. The procedure is referred to as the resistance-to-solar-irradiation test or the simply artificial aging test. It consists of irradiating the lenses of sunglasses with an ozone-free xenon arc lamp (450 W) using a cutoff filter (clear white crown glass B 270; 4 mm thick) between the lamp and the lenses under test, which are placed 300 mm away from the lamp. The lenses are subjected to artificial solar irradiation by the solar simulator for 50 ± 0.1 h [10, 12]. Following the exposure to radiation, spectrophotometry is performed to determine the sunglasses’ transmittance of radiation in the UV-A and UV-B ranges; then, these measurements are compared with the values found before the resistance-to-irradiation test. Thus, the extent to which the UV filters are deteriorated during the aging process can be estimated.

The aim of this test is to establish a correlation between the periods of exposure to natural and simulated sunlight required by many standards for sunglasses. Furthermore, typical periods of exposure are considered based on data obtained from a national survey [2] in Brazil. This correlation varies among different countries and even among different locations within the same country, such as in Brazil. Attempts to match artificial aging tests with environmental counterparts have been problematic in many areas [17–20]. To the best of our knowledge, this is a pioneering effort to achieve such equivalence, at least for sunglasses standards.

Therefore, the objective of whole project is to establish the equivalence between solar exposure during use of the sunglasses and the solar simulator parameters used to carry out the resistance-to-solar irradiation test. Hence, the goal is to provide additional information regarding the parameters used in the UV testing of solar lenses to contribute to the further optimization of the Brazilian standard. Other national standards may also benefit from the present work, especially those nations that are located at similar absolute latitudes.

## Methods

The task of determining the global irradiance on the earth’s surface involves calculations of direct and diffuse solar irradiance. The geometry taken into account in this work refers to an individual who is standing up and wearing sunglasses. In this case, the direct beam irradiance is incident upon a vertical (plane) surface, with a well-known dependence on the incident angle with the normal direction to the surface, described by Lambert’s cosine law. The diffuse irradiance refers to the radiation scattered from the clouds and the atmosphere as well as from the ground and its surroundings.

The starting point in this calculation is to determine the spectral irradiance (in W m^−2^ nm^−1^), called *E*(λ, **r**, *t*), at site level, where λ is the wavelength, **r** collectively represents all spatial coordinates, i.e., geographical position and altitude, and *t* is time of day. For this calculation, we use the SMARTS2 spectral model, proposed by Gueymard [21], which is free to download. The accuracy of this model has been assessed in the literature [21, 22]. The model uses the extraterrestrial solar spectrum (based on satellite data) and through radiative transfer models of the atmosphere, the spectral irradiance is determined at ground level. The model is capable of calculating the direct and the diffuse radiation components for any plane orientation. Specifically, for a vertical plane orientation, the cosine of the incident angle with the horizontal has to be included (oblique incidence). Alternatively, the sine of the zenith angle of solar rays may be used. The sum of the two components is the global irradiance. Thus, the global spectral irradiance can be expressed in the following form:

1 $$ E(\lambda ,{\mathbf{r}},t) = E_{b} (\lambda ,{\mathbf{r}},t)\sin [\theta_{z} ({\mathbf{r}},t)] + E_{d} (\lambda ,{\mathbf{r}},t), $$

where indexes *b* and *d* represent *direct* and *diffuse*, respectively, and *θ*_z_(**r**,* t*) is the zenith angle of the solar beams.

Integration over the appropriate wavelength range yields the solar irradiance *E*(**r**,* t*) (in W∙m^−2^) in terms of the spectral irradiance *E*(λ,** r**, *t*) [Eq. (1)], as follows:

2 $$ E({\mathbf{r}},t) = \int_{{\lambda_{i} }}^{{\lambda_{f} }} {E(\lambda ,{\mathbf{r}},t)\,d\lambda } . $$

Therefore, the radiant exposure (in J·m^−2^) on a surface over a given period is calculated by integrating the irradiance *E*(**r**,*t*) over time, i.e.,

3 $$ H({\mathbf{r}}) = \int_{{t_{i} }}^{{t_{f} }} {E({\mathbf{r}},t)\,dt} . $$

To establish the equivalence between solar radiant exposure (3) and the radiant exposure emitted by a simulator lamp, we calculate the radiant exposure from the lamp using the above-mentioned equations, using the lamp’s spectral irradiance provided by the manufacturer instead of the solar spectral irradiance. Hence, the solar radiant exposure can be compared with the lamp’s radiant exposure.

The fundamental idea is to compute the lamp’s radiant exposure [Eq. (3)] incident on the lenses within the simulator and the sun’s radiant exposure, both in the region 280–492 nm, and compare them with each other. When calculating the lamp’s radiant exposure, one must consider the distance of the samples from the bulb. On the other hand, for the sun’s radiant exposure, the calculation is more difficult due to many variables to be considered. Evidently, the solar irradiance changes during the day and throughout the year at each location, and it is primarily latitude dependent. Thus, we formulate three specific situations for solar irradiance to model, which are quite representative of the conditions that sunglasses are submitted to, as they are worn by an individual throughout a year. In each situation, a different amount of daily hours for wearing sunglasses is considered. Therefore, a daily average of the solar radiant exposure is obtained for each scenario and compared with the lamp’s radiant exposure. The ratio between both expresses a lamp–sun equivalence in “days of use” for each scenario. In other words, for instance, 1 h of exposure in the solar simulator is equivalent to different amount of exposure hours under different solar irradiance conditions, such as the scenarios previously described.

A variety of assumptions, pertaining to both the solar simulator setup and the outdoor environment, can be taken into account to determine this equivalence relation. Those assumptions will be presented and discussed in the following section. In all of that, the oblique incidence (cosine corrected) will be accounted for.

## Results and discussion

Calculations were carried out for the 27 Brazilian state capitals, which span all over the country, and for the specific town of São Paulo, São Paulo State, Brazil, which is a representative example for our purposes. São Paulo is the largest city in Brazil, with nearly 12 million inhabitants, located at latitude −23°32′51″ S, longitude −46°38′10″ W at an average altitude of 760 m. For the northern hemisphere readers, this latitude is approximately equivalent to the latitude of Havana, Cuba. The latitudes of the 27 Brazilian state capitals range from +2°49′11″ N down to −30°01′59″ S. Although our main calculations are performed for Brazilian cities, in fact, other southern hemisphere countries, which share same latitudes, would benefit from our results once those calculations are latitude driven. We also present results for 110 Northern Hemisphere national capitals once many of them are at higher latitudes than nations in Southern Hemisphere. The SMARTS2 model herein used [21], aside information about site location, date, and time, requires input parameters to characterize the atmosphere, such as ozone column, aerosols, turbidity, and others. In addition, it is also possible to input parameters which characterize the local environment, such as soil reflectance. Regarding the atmosphere, for Brazilian cities calculations we have selected the SMARTS2 built-in Tropical standard atmosphere, which has average typical gas concentrations and no pollutants. Likewise, for northern national capitals, we used the SMARTS2 built-in Mid Latitude standard atmosphere. In both cases, the local environment was mainly assumed as urban area with concrete soil. A clear sky assumption has also been made.

Spectral irradiance data corresponding to a distance of 500 mm from the lamp’s bulb (XBO450–OFR xenon arc lamp) were provided by OSRAM over the range 280–2400 nm. Although values of the solar spectral irradiance are available up to a wavelength of 4000 nm, all calculations were carried out over the range 280–492 nm, both for sun and lamp spectral irradiances [see integration limits in Eq. (2)]. The reason for this choice is that this is the range of the fading action spectra, which is primarily in the UV region and, to a lesser extent, in the blue region, corresponding to short wavelength radiation. Moreover, it plays an important role for the ocular health.

The standardized solar irradiance for air mass 1 (AM1) is 1000 W m^−2^, which is expressed as 1 sun. This is the approximate solar irradiance at the Earth’s surface on a horizontal plane at sea level on a clear day, with sun at zenith. Table 1 presents the calculated irradiance of the XBO450–OFR xenon arc lamp from OSRAM for several distances from the lamp bulb for orthogonal irradiation. The sun-equivalent irradiance was calculated as the ratio between the lamp’s irradiance and the standardized solar irradiance (1000 W m^−2^) at each desired distance. The lamp’s spectral irradiance was derived for the desired distances using the inverse square law for point-like light sources. Because the xenon arc length in this lamp is 2.7 mm, according to the manufacturer, a distance from the arc equivalent to five times its largest dimension provides a deviation of 1 % from the inverse square law [23]. In Table 1, the minimum distance from the tested lenses to the lamp used for calculations is 50 mm. For this particular distance, or shorter distances, the extension of the lenses to be irradiated should be taken into account, once the light incidence at the edges of the lenses is not orthogonal. Nevertheless, the standard requires transmittance measurements in a circle of 5 mm radius, centered on the optical axis of the lenses. This requirement ensures a nearly normal incidence in the region of interest, with a maximum deviation of order of 6 % from normal incidence. Therefore, for every distance longer than 50 mm from the bulb, the inverse square law remains valid.

**Table 1:** Lamp (XBO450–OFR) irradiance as a function of the distance *d* (mm) from the lamp bulb and its equivalence in number of suns for AM1

XBO450—OFR OSRAM irradiance (W m^−2^ )	Distance from the bulb *d* (mm)	Equivalent number of suns for AM1
467	300	0.5
672	250	0.7
1000	205	1.0
1051	200	1.1
1868	150	1.9
4202	100	4.2
16,808	50	16.8

1 sun (AM1) = 1000 W m^−2^

It is worth noting that when sunglasses are irradiated 300 mm away from the lamp’s bulb, as required by the standards NBR ISO 12312-1:2015 [10, 11], EN ISO 12312-1:2015 [12], and ISO 12312-1 [13], the equivalent sun irradiance is 0.5, as listed in the first row of Table 1. In other words, the irradiance is similar to that observed when sunglasses are orthogonally exposed to 50 % of the solar irradiance at AM1. The remaining data in Table 1 present the equivalent lamp–sun irradiance values for decreasing distances between the sunglasses and the lamp. Because the inverse square law was used to convert the lamp’s irradiance at 500 mm to that at a desired distance, it should be noted that when the distance is halved, the irradiance is quadrupled. To achieve an exact match between the lamp’s irradiance and one equivalent sun at AM1, the distance from the bulb should be 205 mm.

Brazilian standard [10] and Australian/New Zealand standard [11] require that sunglasses should be irradiated for 50 uninterrupted hours at a distance of 300 mm from the lamp’s bulb in the resistance-to-radiation test. Reasons for that particular distance and period seem unclear and likely lost in history. Under these conditions, according to Table 1, 1 h of lamp exposure is equivalent to 0.5 h of orthogonal sun exposure at AM1, i.e., this simulation system is equivalent to 0.5 sun. Therefore, irradiating sunglasses for 50 h under a simulator should be equivalent to exposing the sunglasses to the sun for 25 h at AM1. This result is not realistic because the atmospheric path of solar beams varies with solar displacement. In addition, it should be considered that when an individual wears sunglasses, the lenses are not orthogonally exposed to the sun because they are usually worn in the vertical position, in which the lenses are not orthogonal to the sun’s rays. Therefore, the incidence angles of solar rays with respect to the sunglasses lenses are relevant, and the sun’s elevation should thus be taken into account, i.e., one should account for oblique incidence.

Some researchers have shown the personal effects of outdoor solar exposure [24, 25] addressing the dermatological aspects.

In this sense, concerns regarding solar exposure are pertinent and the effectiveness of solar simulation on the standards and its parameters are relevant. In order to establish the correspondence of solar simulator and natural sun exposure on sunglasses worn by an individual, some pertinent considerations, named boundary conditions are required.

On authors’ public on-going web survey, 55,000 people have already answered the questions and as a result, most users in Brazil wears sunglasses for at least 2–4 h a day, and purchase new ones every 2 years.

Therefore, three possible scenarios are reasonable to be explored to set a correspondence of sun simulation on sunglasses and natural sun exposure with boundary conditions.

In a recent publication [2], the authors showed that the profile of solar irradiance on vertical surfaces has two distinctive peaks, which indicate the highest irradiances at a given time of day. One of the peaks refers to the time equivalent to the middle of the morning period (average of 143 min after sunrises); the second peak refers to the middle of the afternoon period (average of 143 min before sunsets). Using the established irradiance profiles, three scenarios of solar exposure were analyzed: (1) Sunglasses exposed to the sun over the period spanning from 30 min before the first peak (sunrise in the morning) to 30 min after the second peak, before sunset. The precise time at which each peak occurs shifts throughout the year, and this drift is accounted for. Hence, for each day, the period of exposure to the sun is different. For our purposes, the exposure period is called photoperiod; (2) The photoperiod spanning from sunrise to sunset. This range corresponds to the maximum possible irradiation from the sun and is included herein for comparison purposes; This second scenario, apparently unreal, is quite important for outdoor workers, especially in tropical countries, where a large part of the population is outdoor worker. (3) The 60 min of exposure time centered at the morning peak.

We note that in the three scenarios considered in this work, sunglasses were assumed to be worn in the upright position, tracking the position of the sun and accounting for the oblique incidence. One may argue that, on a daily basis, although the assumption of a vertical position is accurate, the tracking of the sun may be not. The latter assumption can be relaxed by assuming a random vertical positioning of the sunglasses. In this case, the sunglasses are, on average, facing the sun for half of the wearing period, and in the other half, they are worn with the lenses directed away from the sun. Hence, the incident radiant exposure onto the sunglasses is 50 % of the previously calculated amount. Thus, our proposed times for the stress test could be halved.

Also, actual human exposure conditions can be less than our worst-case assumptions, but reduction of UV by automotive windscreens, shading, etc. are not experienced by many who only wear their sunglasses in open environments, e.g., beachgoers, lifeguards, farmers, and most outdoor workers.

### Aging test

For lenses irradiated for 50 h at a distance of 300 mm from the lamp during the aging test, the accumulated radiant exposure [Eq. (3)] delivered by the lamp to the lenses is 7.8 MJ m^−2^.

Comparisons of the lamp’s radiant exposure with the sun’s radiant exposure in the three chosen scenarios were made based on these conditions. In this work, the authors also considered that the sunglasses faced the sun, vertically (with the sunglasses positioned on the face of an individual), for the entire period. For each scenario, we selected a southern summer day (day 355) and a winter day (day 172) to compare the radiant exposure levels. Obviously, those seasons are reversed for Northern Hemisphere. The chosen days represent the solstices, i.e., the year’s longest and shortest photoperiods, because similar to the reason for selecting a position in which sunglasses face the sun for the entire test period, these days provide the most extreme conditions. In addition, the sun’s daily average radiant exposure is herein presented. The daily average was calculated by summing the solar radiant exposure over the entire year and dividing it by 365.25 days. Last column of Table 2 presents the results of the lamp–sun equivalence for each scenario, in which the lamp–sunglasses distance is 300 mm, as established by the standards. The equivalences in “days of use” presented in the last column of Table 2 are determined by the ratio between the lamp’s radiant exposure (6th column) and the global solar radiant exposure (5th column), both italicized for clarity.

**Table 2:** Comparison between the daily solar radiant exposure in São Paulo (SP), Brazil, and the radiant exposure provided by the lamp over a 50-h period (distance between sunglasses and lamp is 300 mm) for 2 specific days of the year: the shortest (day 172) and longest (day 355) days

	Radiant exposure (MJ m^−2^)				Photoperiod (h)	Lamp–sun equivalence (days)
	Solar		Lamp			
	Direct	Diffuse	Global	Direct		
From peak to peak						
Day 172	1.5	0.5	2.0	7.8	4.0	4
Day 355	2.3	1.4	3.7	7.8	8.6	2
Daily average	2.1	1.0	3.2	7.8	6.9	2
Lamp					50.0	
From sunrise to sunset						
Day 172	3.0	1.0	4.0	7.8	10.7	2
Day 355	3.3	1.7	5.0	7.8	13.6	2
Daily average	3.2	1.4	4.6	7.8	12.1	2
Lamp					50.0	
First band peak only						
Day 172	0.4	0.1	0.5	7.8	1.0	16
Day 355	0.4	0.1	0.5	7.8	1.0	16
Daily average	0.4	0.1	0.5	7.8	1.0	16
Lamp					50.0	

In addition, the daily average is shown

In the first scenario, sunglasses were exposed to solar radiation from half an hour before the first peak in the direct solar radiant exposure profile up to half an hour past the second peak for a particular day. In this scenario, the global solar radiant exposure, which is the sum of the direct and diffuse components, amounts to 3.7 MJ m^−2^ for day 355 (southern summer day). Hence, the lamp’s radiant exposure (over a 50-h period), which sums to 7.8 MJ m^−2^, is two times greater than the solar radiant exposure of day 355 (see second row in Table 2). Thus, the exposure time of 50 h in the simulator is equivalent to the exposure to sunlight for approximately 2 days of specific day 355. In this scenario, day 355 has 8.6 h (from peak to peak) of exposure time to sunlight. Therefore, the national standard requirements for aging tests—in which lenses are exposed for 50 h to a 450 W lamp (XBO450–OFR) at a distance of 300 mm from the lamp bulb—appears to be inadequate for aging tests, at least with regard to the superficial radiant exposure equivalence between the exposure to the lamp and to the natural environment.

Even for a less severe scenario, such as exposure on a winter day (e.g., day 172, for southern hemisphere), the solar radiant exposure components that reach a vertical surface are 1.5 MJ m^−2^ (direct) and 0.5 MJ m^−2^ (diffuse), resulting in a global radiant exposure of 2.0 MJ m^−2^. Assuming the same testing conditions described previously, the lamp-exposure time (50 h) is equivalent to 4 days (the photoperiod for day 172 is 4.0 h). Once more, the requirements defined for the aging tests are not sufficient.

Calculations were performed for each day of the year to allow the results to be averaged throughout the year, yielding a daily average. Table 2 summarizes the average results alongside the results for the particular days referenced above. The table also presents a comparison with results obtained for the entire photoperiod of each day, i.e., from sunrise to sunset.

Table 2 presents the central results of this work. It can be observed that the test for sunglasses’ resistance to radiation (and the aging process thereof) required by the standards only probes the deterioration of the UV protection of the lenses for quite a short period and is therefore insufficient to guarantee their safety in terms of eye protection. Thus, the solarization test is ineffective and has no practical value.

To overcome these limitations of the standard requirements, one may either increase the exposure time of the lenses to the lamp or decrease the distance of the lenses from the lamp. Increasing the exposure time is certainly possible, although doing so may increase the cost and certification time, eventually causing the procedure to become impractical. According to Table 1, decreasing the distance from the lamp may be a more effective alternative because of the inverse square law for point sources. For instance, setting the distance from the lamp to 50 mm yields the results presented in Table 3. As expected, a sixfold reduction in distance increases the lamp–sun equivalence to a factor of 36, compared with values presented in last column of Table 2. On the other hand, increasing the exposure times avoids the consequential temperature rise that may come from decreasing the distance. A third alternative would be to change the 450 W lamp to higher power lamp, e.g., a 1600 W lamp, which is commercially available. However, this would require a major evaluation of this requirement in the standards, especially the specifications of the simulator as a whole.

**Table 3:** Comparison between the daily solar radiant exposure in São Paulo (SP), Brazil, and the radiant exposure provided by the lamp over a 50-h period (distance between sunglasses and lamp is 50 mm) for 2 specific days of the year: the shortest (day 172) and longest (day 355) days

		Radiant exposure (MJ m^−2^)		Lamp–sun equivalence (days)
		Solar	Lamp	
		Global	Direct	
1. From peak to peak	Day 172	*2.0*	*280.3*	140
	Day 355	*3.7*	*280.3*	76
	Daily average	*3.2*	*280.3*	88
2. From sunrise to sunset	Day 172	*4.0*	*280.3*	70
	Day 355	*5.0*	*280.3*	56
	Daily average	*4.6*	*280.3*	61
3. First band peak only	Day 172	*0.5*	*280.3*	561
	Day 355	*0.5*	*280.3*	561
	Daily average	*0.5*	*280.3*	561

In addition, the daily average is shown

Based on informed estimates, it is quite reasonable to assume that the UV protection of sunglasses should be required to last at least 2 years (730.5 days) under the first scenario considered in this work. To simulate such a case, simply decreasing the distance from the lamp in the stress tests is insufficient, and the exposure time must be increased. For instance, on third row in Table 3, at lamp-sunglasses distance of 50 mm, the lamp provides 280.3 MJ m^−2^ for the 50 h of simulation period. Under the assumptions of the first scenario, the solar radiant exposure is, in average, 3.2 MJ m^−2^ per day. Thus, the ratio lamp–sun is 88 days. Hence, to increase the lamp–sun equivalence from 88 days to 730.5 days (2 years), the total radiant exposure of the lamp should be increased by a factor of 8.3, i.e., from 280.3 MJ m^−2^ to 2326.5 M m^−2^. This means to increase the period of the 450 W lamp simulator by the same factor, i.e., from 50 to 414.6 h of exposure, at a distance of 50 mm.

To simulate the unlikely scenario of an individual who wears sunglasses from sunrise to sunset (in São Paulo, Brazil), the lamp–sun equivalence should be increased even more, and the lamp-exposure time should be increased to 603.7 h.

Table 4 presents the calculated data for radiant exposure lamp–sun equivalence, in days, for decreasing distances between the lamp and tested sunglasses. The data were calculated for 27 state capitals in Brazil. For each scenario and particular distance, the minimum and maximum values are listed. The entries labeled MED in Table 4 are the median values among all 27 locations in Brazil for which the calculations were carried out. Once the latitude distribution of all locations considered in this work is non uniform, the median was calculated instead of the average to avoid unintended deviations. As expected, the lamp–sun equivalences as functions of distance, shown in each row of Table 4, follow an inverse square law.

**Table 4:** Calculated radiant exposure lamp–sun equivalences (in “days of use”) for different scenarios and for a decreasing distance *d* (mm) between the lamp and sunglasses. The minimum and maximum lamp–sun equivalences are listed

	Distance (*d*) from lamp (mm)					
	300	250	200	150	100	50
1. From peak to peak						
Min	2	3	5	9	21	83
Max	3	4	6	11	26	103
Med	2	3	5	9	21	84
2. From sunrise to sunset						
Min	2	2	4	7	15	60
Max	2	3	4	7	16	62
Med	2	3	4	7	16	62
3. First band peak only						
Min	15	22	34	60	134	537
Max	15	22	35	62	139	556
Med	15	22	35	62	139	555

Additionally, the medians of all 27 cities are shown

Evidently, a typical person wears sunglasses throughout the year over a period of less than 8–12 h a day on average (our survey [2] indicates an average of 2 h daily). In such cases, the user may wear his/her sunglasses over a longer season while retaining the UV protection of the lenses. Tables 2, 3 and 4 present results calculated for the third scenario, in which an individual wears sunglasses for a typical period of 1 h daily when this period is assumed to coincide with the maximum exposure to solar radiation. To simulate this case, the lamp-exposure time should be 67.3 h (at a distance of 50 mm) to ensure a protection lifetime of 2 years (730.5 days).

Based on the survey of the Brazilian population, most users wears the same pair of sunglasses for a minimum of 2 years and for a period of 2 h a day. Therefore, the standard must guarantee that sunglasses should be safe over this period. In this case, the solarization test should be performed for 134.6 h (at a distance of 50 mm). In this respect, our contribution is the refinement of the parameters required by current standards for solar simulator exposure.

In order to extend the scope of this work, Table 5 presents, similarly, the same results as Table 4 for 110 national capitals from Northern Hemisphere. It is worth noting that the results for the lamp–sun equivalences are very similar to the values from Brazil, with a slight difference in favor of Norther Hemisphere due to the higher latitudes in general. Nevertheless, the results indicates that the solarization test of sunglasses is inadequate even for countries in Northern Hemisphere.

**Table 5:** Calculated radiant exposure lamp–sun equivalences (in “days of use”) for different scenarios and for a decreasing distance *d* (mm) between the lamp and sunglasses

	Distance (*d*) from lamp (mm)					
	300	250	200	150	100	50
From peak to peak						
Min	2	3	5	9	19	78
Max	7	10	16	28	63	252
Med	3	4	7	12	27	107
From sunrise to sunset						
Min	2	2	4	6	14	57
Max	2	3	5	9	21	84
Med	2	3	4	7	16	62
First band peak only						
Min	14	21	32	57	129	516
Max	23	33	52	93	209	836
Med	16	22	35	62	139	557

The minimum and maximum lamp–sun equivalences are listed. Additionally, the medians of all 110 national capitals from northern hemisphere are shown

As in Brazil the sun delivers 0.5 MJ m^−2^ a day, for the third scenario, in 24 months, it would be delivered an amount of 365.3 M m^−2^ (0.5 M m^−2^ × 730.5 days). Therefore, for implementing such requirement for the “resistance to radiation test” of the standards, an appropriate solar simulator, which provides irradiance, should be architected in order to supply accelerated simulation of sun exposure. It should assemble adequate lamp power, exposure time, distance from the bulb and controlled temperature that the sample will be exposed to.

## Conclusions

The present test parameters for exposing samples to a solar simulator, as specified by the Brazilian and many national standards, should be revisited to establish safe limits for UV filters of sunglasses. By changing the exposure time within the solar simulator and the distance of the samples from the lamp, respectively, to 67.3 h and 50 mm, sunglasses can be safe to wear for a period of 2 years for users who wear them for a maximum of 2 h a day. It is worth noting that the temperature inside a solar simulator should not exceed limits that deteriorate the optical properties of sunglasses. Thus, it has to be assured by further investigation that the temperature inside the solar simulator at this distance from the lamp does not reach inappropriate levels.

Our calculations were made to ensure the safety of sunglasses worn in Brazil, but are also valuable to countries that share same latitudes. Additionally, results for 110 national capitals in northern hemisphere were presented, broadening the reach of this effort to help establish safe limits for UV filters of sunglasses.

## Abbreviations

UV: ultraviolet
ICNIRP: International Commission on Non-Ionizing Radiation Protection
CIE: International Commission on Illumination (Commission Internationale de l’Eclairage)
SMARTS2: simple model of the atmospheric radiative transfer of sunshine v. 2
AM: air mass
